# Effect of Nifedipine in Preventing Ovarian Hyperstimulation Syndrome through TRPC1 Ion Channel Inhibition

**DOI:** 10.1007/s43032-025-01913-8

**Published:** 2025-06-18

**Authors:** Emel Kocal, Remzi Atilgan, Şehmus Pala, Melike Aslan, Tuncay Kuloğlu, Nevin Ilhan, Ebru Etem Önalan, Serhat Hançer, Gizem Kaymaz Bircan

**Affiliations:** 1 Department of Obstetrics and Gynecology, Firat University School of Medicine, Elazig, Turkey; 2 Department of Histology and Embriology, Firat University School of Medicine, Elazig, Turkey; 3 Department of Biochemistry, University School of Medicine, Elazig, Turkey; 4 Department of Medical Biology, Firat University School of Medicine, Elazig, Turkey

**Keywords:** OHSS, Rat, Nifedipine, TRPC1, VEGF

## Abstract

Ovarian hyperstimulation syndrome (OHSS) is a life-threatening complication that usually develops as a result of triggering ovulation with human chorionic gonadotropin (hCG) after gonadotropin treatment, and in whose pathophysiology vascular endothelial growth factor (VEGF) and inflammatory mediators play a role. Nifedipine, used especially in the treatment of hypertension, is a calcium channel blocker. Nifedipine also has anti-inflammatory effects via transient receptor potential canonical (TRPC1) ion channel inhibition. VEGF also regulates the angiogenic process through TRPC channels. In our study, we investigated the potential of nifedipine to prevent OHSS due to its TRPC1 blocking effect and anti-inflammatory effects. A total of 28 rats were randomly divided into four equal groups. Group (G) 1 control group (*n* = 7). Rats in G2 (*n* = 7) were administered 30 IU pregnant mare serum gonadotropin for 4 days and OHSS was induced by administering 30 IU hCG on the fifth day. Rats in G3 (*n* = 7) were induced to have OHSS and were given 100 μg/kg oral cabergoline, while rats in G4 (*n* = 7) were induced to have OHSS and were given 20 mg/kg intraperitoneal nifedipine. On the fifth day, all rats were decapitated and VEGF, interleukin (IL)-1β, IL-6, tumor necrosis factor (TNF)-α, and hypoxia-inducible factor (HIF)-1α levels were measured in their serum and tissues. TRPC1 gene expression and immunohistochemical analysis were performed in ovarian tissue. We showed that nifedipine inhibited VEGF and some inflammatory factor levels more than cabergoline. We showed that nifedipine may achieve these effects through TRPC1 blockade and suppression of inflammatory factors.

## Introduction

Fluid leakage into the third space as a result of increased capillary permeability is the hallmark of OHSS [1], a complication that typically arises as a result of infertility therapies and occasionally has a potentially fatal potential. Histopathological alterations brought on by OHSS in the ovaries include increased luteal follicle cysts, necrosis and neovascularization, edema, and ovarian enlargement [2]. The American Society for Reproductive Medicine (ASRM) classifies OHSS into three categories: mild, moderate, or severe, based on the presence of OHSS symptoms. Moderate to severe OHSS occurs in approximately 1–5% of in vitro fertilization (IVF) cycles with an incidence of up to 20% in high-risk patients [3, 4]. It has been reported that 11,562 cases were hospitalized due to OHSS in the United States from 2002 to 2011, and approximately 4.4% of these cases experienced life-threatening complications [5].

Research has demonstrated a connection between OHSS and elevated vascular permeability as a result of proinflammatory cytokine production brought on by hCG-induced ovulation [6]. Furthermore, it has been demonstrated that vascular endothelial growth factor (VEGF) and a variety of angiogenic substances and cytokines contribute to the development of OHSS [7]. VEGFs are produced by granulosa cells as a result of gonadotropin stimulation, and their production increases significantly after hCG administration. In addition, other systemic and local vasoactive substances, including interleukin (IL)−2, IL-6, IL-8, IL-10, IL-18, angiotensin II, histamine, prolactin, prostaglandins, insulin-like growth factor (IGF) 1, and transforming growth factor (TGF) b, are also directly and indirectly involved in the pathogenesis of OHSS [4, 8–10]. Hypoxia-induced factor-1 alpha (HIF-1α) is an essential transcription factor and plays a critical role in almost all processes of wound healing and vascular remodeling [11, 12]. It has been reported that overexpression of HIF-1α can activate the VEGF/AKT/mTOR signaling pathway by increasing VEGF expression and lead to increased p-AKT and p-mTOR levels [13]. According to some research, reducing vascular permeability—typically by inhibiting VEGF secretion—can stop the development and progression of OHSS [1, 14, 15]. In this work, we examined how the ancient medication nifedipine affected OHSS by inhibiting VEGF secretion via the transient receptor potential canonical (TRPC1) ion channel. Through pro-angiogenic molecules like VEGF and basic fibroblast growth factor, endothelial TRPC channels regulate the angiogenic process by delivering calcium ions [16]. Through VEGF and basic fibroblast growth factor, among other factors, TRPC1 has been demonstrated to control angiogenesis [17]. Nifedipine is a vasoselective calcium channel blocker that inhibits voltage-dependent L-type calcium channels in cells and stops calcium ions from entering cells. It is particularly used to treat hypertension. Oral administration of nifedipine results in nearly total absorption. Nifedipine's main adverse effects include headache, palpitations, burning and redness in the face and legs, and ankle edema from skin vascular dilatation [18–20].

## Materials and Methods

The Fırat University Experimental Animals Research Center conducted this randomized controlled experimental study in compliance with ethical guidelines after receiving approval from the Fırat University, Faculty of Medicine Experimental Animals Ethics Committee on February 27, 2023. The study used 28 female Spraque-Dawley rats that weighed 45–60 g and were 22–23 days old. FÜDAM supplied and provided accommodation for the animals. The rats were housed in specially designed cages with city water and pellet meal, maintained at room temperature between 22 and 25 °C, and exposed to 12 h of light (7:00–19:00) and 12 h of darkness (19:00–7:00). The bottoms of the cages were cleaned daily.

According to the principles of laboratory animal care, all research animals were cared for in accordance with the guidelines for the care and use of animals that our institutions have approved (NIH Guide for the Care and Use of Laboratory Animals, Institute of Laboratory Animal Resources, National Research Council, Washington, D.C.). Twenty-two-day-old female rats (weighing 45–50 g) were randomly divided into 4 groups.

Group (G) 1 (control, *n* = 7) = group given 0.1 ml saline intraperitoneally (ip) for five consecutive days (between days 22 and 26).

G2 (OHSS group, *n* = 7) = 30 IU pregnant mare serum gonadotropin (PMSG) (Folligon®-Intervet; Schering-Plough Animal Health, Pune, India) was given subcutaneously for 4 consecutive days and 30 IU hCG (Chorulon®-Intervet; Schering-Plough Animal Health Boxmeer, The Netherlands) was given on the fifth day to induce severe OHSS (Severe OHSS protocol).

G3 (*n* = 7) = Severe OHSS induced and given 100 μg/kg cabergoline [(Dostinex – Pharmacia and Upjohn SpA, Milan, Italy) dissolved in 5% glucosal] orally on the day of hCG administration [21].

G4 (*n* = 7) = Severe OHSS induced and given 20 mg/kg intraperitoneal nifedipine (Adalat – 20 Tablets, Bayer Healthcare, New Zealand) in saline solution on the day of hCG administration [22].

**Fig. 1:**
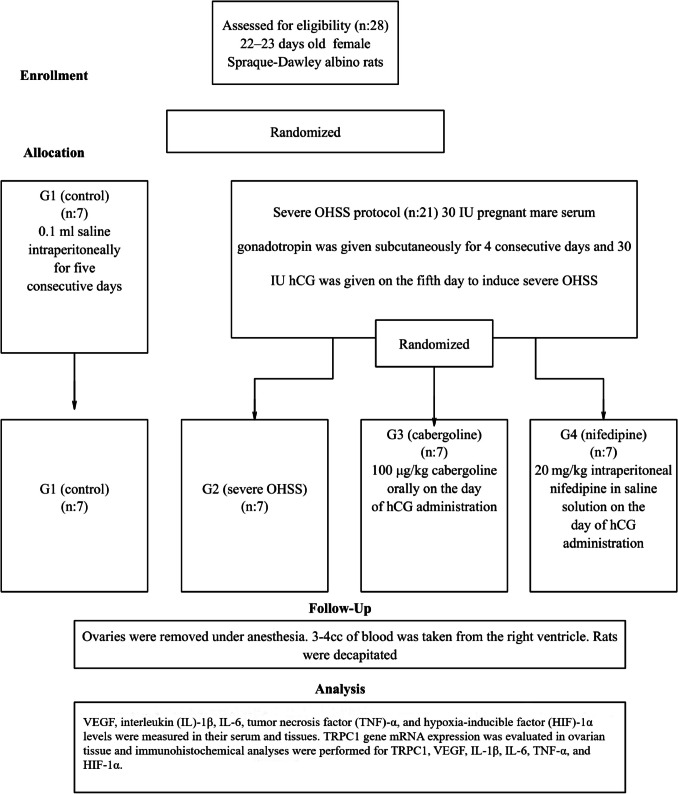
The consort of the experimental study

On the 27th day, the rats were given 80 mg/kg ketamine (Ketalar, Eczacıbaşı, İstanbul, Turkey) and 20 mg/kg xylazine (Rompun Vet, Bayer AB, İstanbul, Turkey) to induce anesthesia and open their abdomens. Following their complete removal, the ovaries were promptly weighed on a precision scale, and their respective weights were noted. Next, 3–4 cc of blood were extracted from each rat's right ventricle and placed in gel biochemistry tubes. High-dose anesthetic was used to put the rats to sleep after the blood collection procedure. Up until the day of the trial, right ovarian tissue was kept at −80 °C for genetic and biochemical analysis. For immunohistochemistry analysis, 10% formaldehyde was used to fix the left ovary tissue, which was then embedded in paraffin blocks. The consort of the experimental study is shown in Fig. 1.

### Immunohistochemical Examination

Sections from paraffin blocks that were 4–6 μm thick were put on polylysine slides. For antigen retrieval, deparaffinized tissues were boiled in a citrate buffer solution at a pH of 6 for 15 min in a 750 W microwave oven after passing through a graded alcohol series. The tissues were boiled and then allowed to cool for about 20 min at room temperature. To stop endogenous peroxidase activity, they were incubated with hydrogen peroxide block solution (Hydrogen Peroxide Block, TA-125-HP, Lab Vision Corporation, USA) for five minutes after being cleaned for 3 × 5 min with PBS (Phosphate Buffered Saline, P4417, Sigma-Aldrich, USA). After 5 min of applying Ultra V Block (TA-125-UB, Lab Vision Corporation, USA) solution to prevent background staining, tissues were incubated with a 1/200 diluted TRPC1, for 60 min at room temperature in a humid environment. Following the primary antibody application, the tissues underwent 3 × 5 min PBS washes before being incubated for 30 min at room temperature in a humid environment with the secondary antibody (biotinylated Goat Anti-Polyvalent (anti-mouse/rabbit IgG), TP-125-BN, Lab Vision Corporation, USA). Tissues were incubated with Streptavidin Peroxidase (TS-125-HR, Lab Vision Corporation, USA) for 30 min at room temperature in a humid environment after the secondary antibody was applied. They were then rinsed with PBS for 3 × 5 min each time. Once the image signal was collected under a light microscope, the tissues were simultaneously washed with PBS and 3-amino-9-ethylcarbazole (AEC) Substrate + AEC Chromogen (AEC Substrate, TA-015 and HAS, AEC Chromogen, TA-002-HAC, Lab Vision Corporation, USA). The tissues were washed with PBS and distilled water, counterstained with Mayer's hematoxylin, and then coated with the proper covering solution (Large Volume Vision Mount, TA-125-UG, Lab Vision Corporation, USA). Under a Leica DM500 microscope, the created preparations were inspected, assessed, and captured on camera (Leica DFC295).

**Fig. 2:**
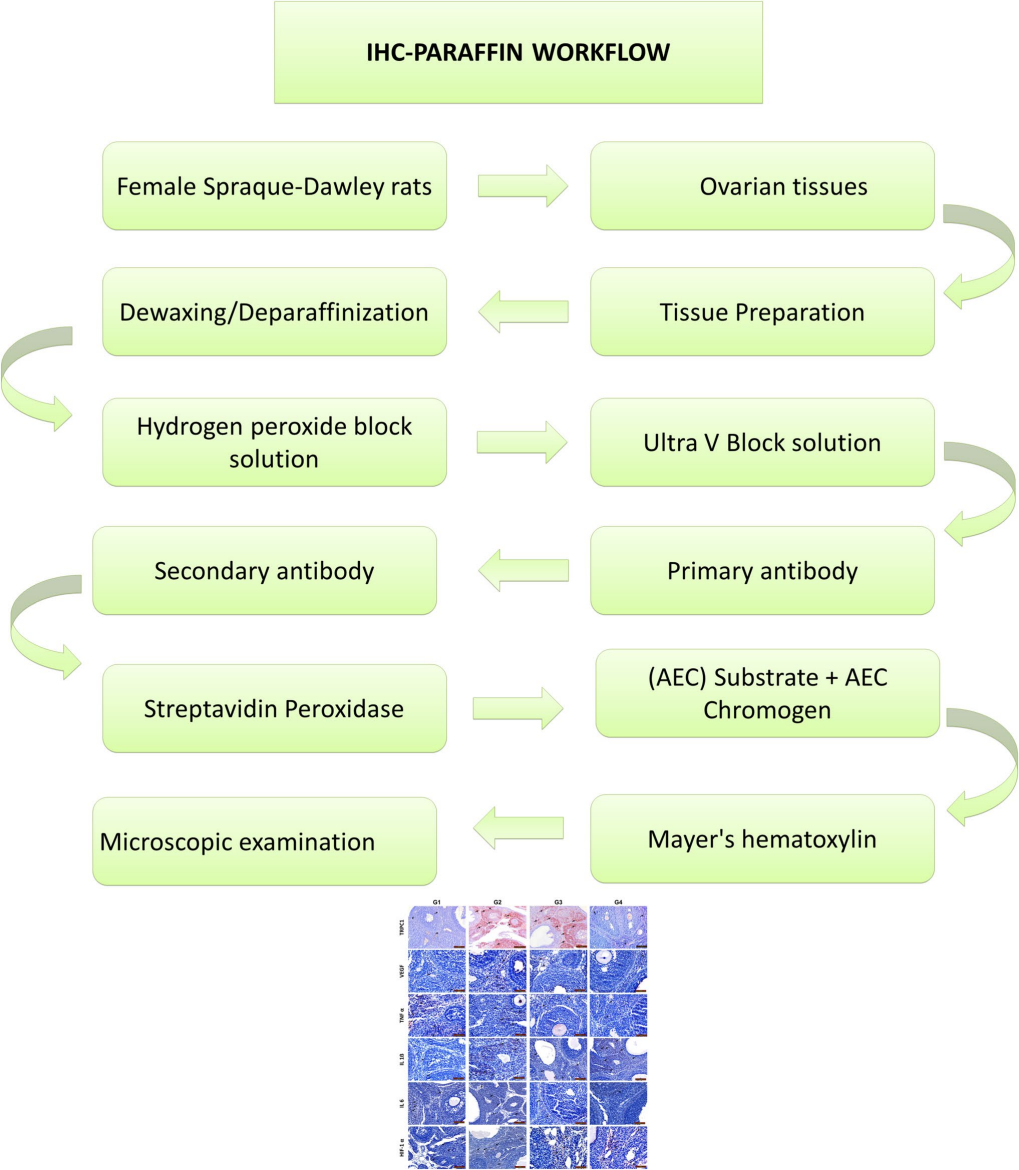
Explanation scheme about immunohistochemistry examination

Histoscore was created based on the prevalence (0.1: < 25%, 0.4: 26–50%, 0.6: 51–75%, 0.9: 76–100%) and severity (0: none, + 0.5: very little, + 1: little, + 2: moderate, + 3: severe) of immunoreactivity in staining. Histoscore = prevalence x severity [6]. The explanation scheme about immunohistochemistry examination is shown in Fig. 2.

### Quantitative Real Time Polymerase Chain Reaction (qRT-PCR)

Variations in the levels of TRPC1 gene mRNA expression in ovarian tissues were found using the qRT-PCR technique. A tissue sample weighing 30–40 mg was isolated using TrizolTM Reagent (Cat no. 15596018, Thermo Fischer Scientific). Thirty microliters of diethylpyrocarbonate (DEPC) water were used to dissolve the resulting RNA pellet. A nanodrop device (BioSpec-nano, Shimadzu) was used to measure the quantities of RNA. The lowest RNA value read was accepted as the standard value to guarantee equal RNA levels in the complementary DNA (cDNA) synthesis stage. A total volume of 10 μL was used for cDNA synthesis, which included 5 μL of RNA sample, 1 μL of 10 × RT Buffer, 0.5 dNTP mix (2.5 mM), 1 μL of Random Hexamer (50 μM), 0.5 μL of Reverse Transcriptase, 0.25 μL of RNase Inhibitor, and 1.75 μL of Nuclease-free H2O.

After placing in the thermal cycler, the samples were maintained at 25 °C for 10 min, 37 °C for 120 min, 85 °C for 5 min, and 4 °C for the final temperature. The samples were placed in the thermal cycler and kept in the device for 10 min at 25 °C, 120 min at 37 °C, 5 min at 85 °C and the final temperature was 4 °C. cDNAs obtained by reverse transcription were used to identify rat-specific β-actin (housekeeping or control gene) with the names Rn-β-actin forward 5ˈ-AGCCATGTACGTAGCCATCC-3ˈ and 5ˈ-TCGGAACCGCTCATTGCCG-3ˈ [23] and 5ˈ-TTC CAA AGA GCA GAA GGA CTG-3ˈ and 5ˈ-AGG TGC CAA TGA ACG AGT G-3ˈ primers for TRPC1 were amplified by qRT-PCR in triplicate [24]. One microliter of cDNA sample, 5 µL of 2 X Magic SYBR Mix (Procomcure Biotech GmbH), 0.5 µL of Forward Primer (Sentegen), 0.5 µL of Reverse Primer (Sentegen), and 3 µL of Nuclease-free H2O were added to each well to generate the qRT-PCR combination. The plate was then covered with optical adhesive film.

Using the Applied Biosystems 7500 RT-PCR system, it was amplified at 95 °C for 5 min, 95 °C for 10 s, 58 °C for 30 s, 72 °C for 30 s, and 72 °C for 2 min. Differences in gene expression resulting from qRT-PCR were calculated using the 2-∆∆CT technique.

### Biochemical Study

Serum was isolated from blood samples that were collected in plain tubes and centrifuged for 10 min at + 4 °C at 4000 rpm. Eppendorf tubes were used to separate the serum samples, which were then kept at −80 °C until the day of analysis. Samples removed from −80 ^0^C were rapidly thawed at 25 ℃ in a shaking water bath (Köttermann labortechnik, type 3047, Germany) and biochemical analyses were performed. After removing the entire ovarian tissue, the tissues (1:9; w:v) were placed in tubes with 0.01 M phosphate buffer (PBS; pH 7.4) and homogenized for 3 min at 4 °C and 16,000 rpm. The resulting homogenates were separated from the supernatants after being centrifuged at 5000xg for 15 min at + 4ºC. The amount of protein in the supernatant was measured by measuring the blue complex produced by proteins at 650 nm using Folin-Phenol reagent in alkaline medium. Using the Enzyme-Linked Immuno Sorbent Assay (ELISA), the levels of VEGF, TNF-α, IL-1β, IL-6, and HIF-1α were assessed in serum and supernatants. Values per milligram of protein were computed from the supernatant results. VEGF, HIF-1α, TNF-α, IL-1β, and IL-6 levels in tissue and serum were examined using the appropriate ELISA kit protocols. The EPOCH 2 microplate reader (BioTek Instrument, Inc., USA) was used to spectrophotometrically read absorbances at 450 nm. The relevant biochemical parameter's unit was used to present the results. Table 1 lists all of the biochemically examined parameters along with the manufacturer, country of origin, catalogue number, kit measurement range, and kit sensitivity.

**Table 1:** Country, company, catalog number, kit measurement range and kit sensitivity of the Enzyme-Linked Immunosorbent Assay (ELISA) kits used in the study

Parameters	Company and country	Catalog number	Measuring range	Sensitivity
VEGF	Sunred Biotechnology Company, Shanghai, China	201–11–0660	11–3000 ng/L	10,127 ng/L
HIF-1 α	ELK Biotechnology, Wuhan, China	ELK1604	0,16–10 ng/mL	0,056 ng/mL
TNF − α	ELK Biotech. Biotechnology Company, Wuhan, China	ELK1396	15.63–1000 pg/mL	6.1 pg/mL
IL 1-β	Sunred Biotechnology Company, Shanghai, China	201–11–0120	25–8000 pg/L	20,118 pg/L
IL-6	Sunred Biotechnology Company, Shanghai, China	201–11–0136	2–600 pg/mL	1,822 pg/mL

*VEGF *vascular endothelial growth factor, *TNF − α *tumor necrosis factor − alpha, *IL − 1β *interleukin − 1 beta, *IL-6 *interleukin − 6, *HIF − 1α *hypoxia-inducible factor − 1 alpha

### Statistical Analysis

All of the data collected for this study were statistically evaluated using the SPSS 22.0 package. Numerical data were expressed as median (minimum—maximum). To determine if the variables were normally distributed, the Shapiro–Wilk test was employed. For general comparisons between more than two groups, the Kruskal Wallis test was employed. Following Kruskal Wallis, the post-hoc Dunn test was employed to compare the two groups. The level of statistical significance was set at *P* < 0.05.

## Results

### TRPC1 Immunoreactivity

**Fig. 3:**
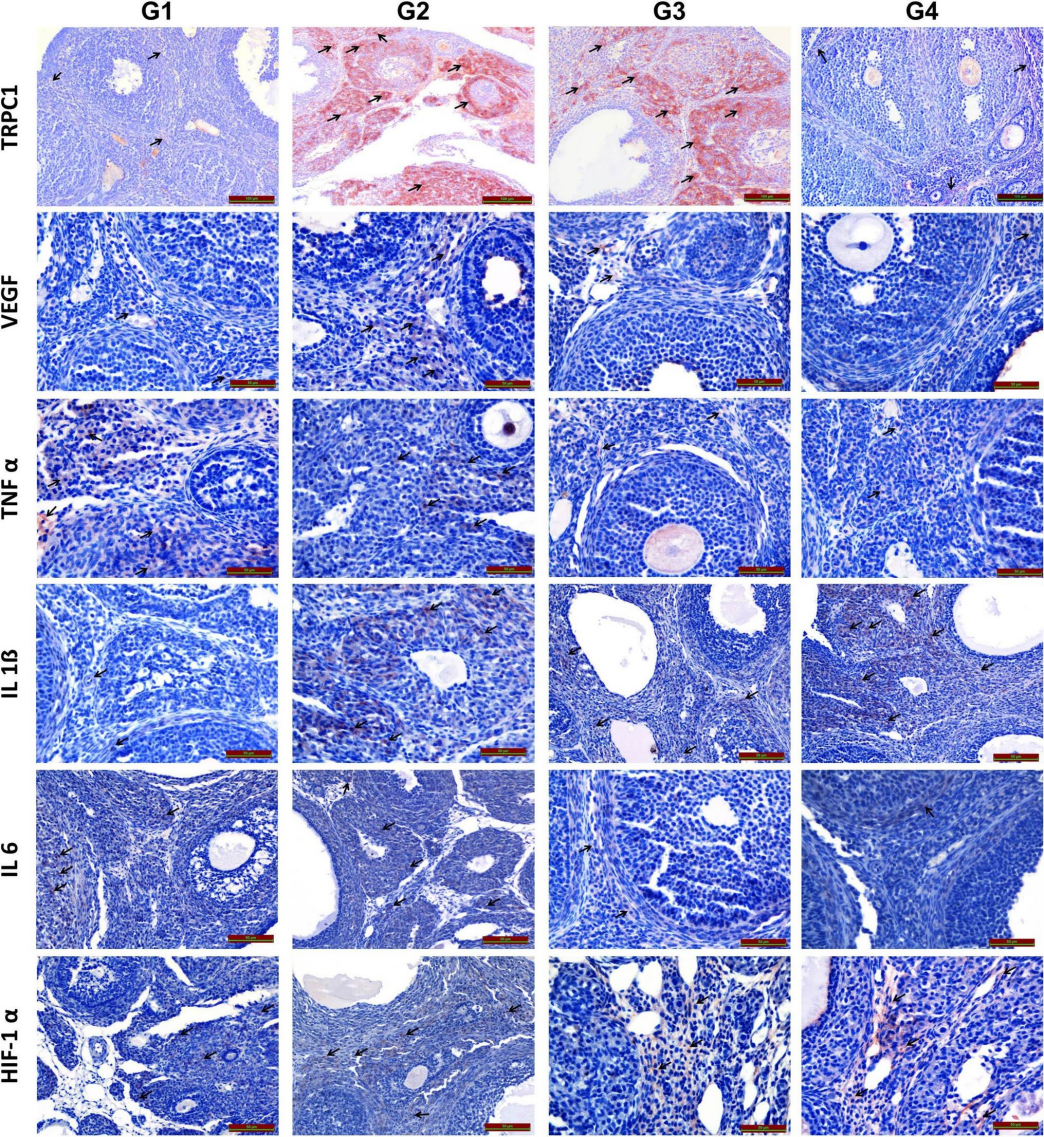
TRPC1, VEGF, HIF-1α, IL-1β, IL-6 and TNF-α immunohistochemical staining. Immunoreactive staining in all groups is shown with black arrows

In comparison to G1, TRPC1 immunoreactivity was statistically significantly higher in G2 (*p* = 0.010) and G3 (*p* = 0.023). Comparing G1 and G4, there was no statistically significant difference in TRPC1 immunoreactivity (*p* = 0.998). TRPC1 immunoreactivity in G3 did not change statistically significantly from that in G2 (*p* = 0.996); however, it did decrease statistically significantly in G4 (*p* = 0.002). However, when comparing G4 to G3, there was a statistically significant reduction in TRPC1 immunoreactivity (*p* = 0.006), (Table 2), (Fig. 3).

**Table 2:** TRPC1 TRPC1 immunoreactivity histoscore in the ovarian tissue (Values ​​are given as median, minimum–maximum)

Groups	TRPC1 immunoreactivity histoscore
G1	0,40 (0,30–0,45)
G2	1,20 (0,90–2,70)^a^
G3	1,20 (0,80–1,80)^a^
G4	0,40 (0,20–0,60)^bc^
P*	< 0,001

^*^Kruskal–Wallis

^a^ Compared with G1

^b^ Compared with G2

^c^ Compared with G3, (*p* < 0.05)

### Genetic Findings

**Fig. 4:**
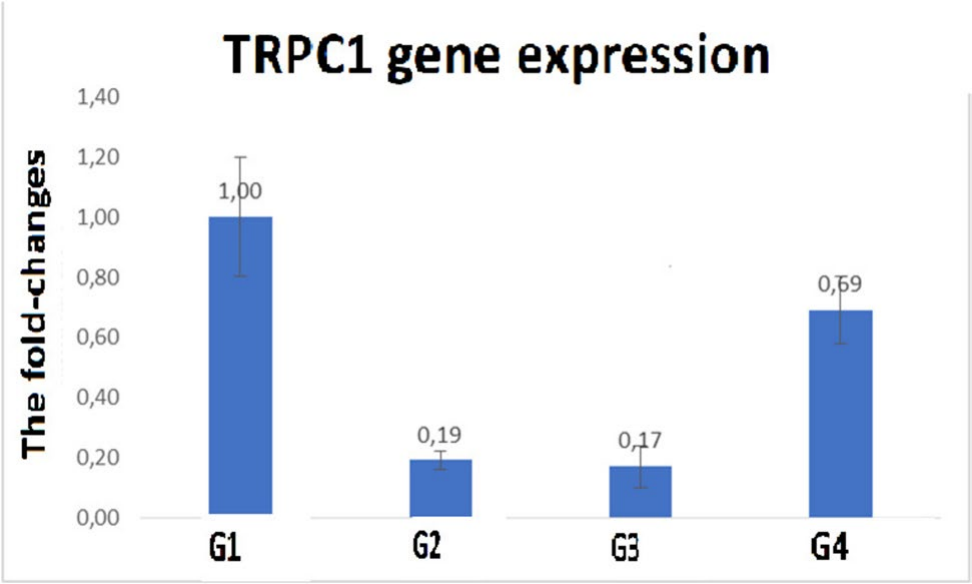
TRPC1 gene expression was shown to be significantly decreased in OHSS (G1) (*p*=0.009), cabergalin application (G3) could not correct this decrease (comparing G1 and G3; *p*=0.008), but nifedipine application (G4) significantly increased TRPC1 expression compared to G2 and G3 (*p*=0.007 and *p*=0.008). Data are expressed as mean ± SEM. Significant difference compared to the G1 at ap<0.05, compared to G2 at bp<0.05 and compared to G3 at cp<0.05. Abbreviations: TRPC1: transient receptor potential canonical; VEGF: vascular endothelial growth factor; HIF-1 α: hypoxia-inducible factor-1 alpha; IL-1β: interleukin-1 beta; IL-6: interleukin-6; TNF-α: tumor necrosis factor alpha; G1: Control group; G2: OHSS group; G3: OHSS group given cabergoline; G4: OHSS group given nifedipine

The administration of cabergoline did not reverse the significant decrease in TRPC1 gene expression that was observed as a result of OHSS exposure (G1 vs. G2; *p* = 0.009), while the administration of nifedipine significantly increased TRPC1 expression in comparison to G2 and G3 (*p* = 0.007 and *p* = 0.008). Figure 4 displays the qRT-PCR fold change graph. These findings demonstrate that whereas nifedipine administration corrected the decline in TRPC1 levels brought on by OHSS, cabergoline administration did not.

### Biochemical Findings

#### Serum VEGF Levels

Serum VEGF levels in G2 were found to be statistically significantly higher than those in G1 (p < 0.007). Serum VEGF levels were shown to be statistically significantly lower in G3 (*p* = 0.011) and G4 (*p* = 0.008) than in G2. Nevertheless, there was no noticeable statistically significant difference between G3 and G4 (*p* = 0.935), (Table 3).

**Table 3:** Serum VEGF, HIF-1 α, IL-1ẞ, IL-6 and TNF-α levels in all groups (Values ​​are given as median, minimum–maximum)

Parametre	G1	G2	G3	G4	P*
VEGF (pg/ml)	4,52 (1,18–7,27)	55 (21–90)^a^	4,72 (1,25–8,42)^b^	4,18 (2,24–6,98)^b^	0,002
HIF-1 α (pg/ml)	0,187 (0,18–3,25)	0,184 (0,17–4,72)	0,217 (0,18–7,33)	0,178 (0,16–8,54)	0,597
IL-1ẞ (pg/ml)	328 (274–902)	5268 (1257–9498)^a^	1077 (755–1992)	1059 (214–1196)^b^	< 0,001
IL 6 (pg/ml)	2,550 (0,37–59,19)	87,037 (83,78–126)^a^	69 (1,42–92)	2,074 (0,63–12,14)^b^	0,001
TNF-α (pg/ml)	208 (159–341)	516 (102–425)^a^	285 (196–408)	201 (71–392)^b^	0,001

*VEGF *vascular endothelial growth factor, *TNF − α *tumor necrosis factor − alpha, *IL − 1β *interleukin − 1 beta, *IL-6 *interleukin − 1, *HIF − 1α *hypoxia-inducible factor − 1 alpha, *G1 *Control group, *G2 *OHSS group, *G3 *OHSS group given cabergoline, *G4 *OHSS group given nifedipine

^*^Kruskal–Wallis

^a^ Compared with G1

^b^ Compared with G2, (*p* < 0.05)

#### Tissue VEGF Levels

Tissue VEGF levels in G2 were statistically significantly higher than those in G1 (*p* = 0.003). In comparison to G2, tissue VEGF levels were statistically significantly lower in the G3 (*p* = 0.046) and G4 (*p* = 0.01) groups. G3 and G4 did not, however, differ statistically significantly (*p* = 0.616), (Table 4).

**Table 4:** Ovarian tissue VEGF, HIF-1 α, IL-1ẞ, IL-6 and TNF-α levels in all groups (Values ​​are given as median, minimum–maximum)

Parameters	G1	G2	G3	G4	P*
VEGF (pg/ml)	2,066 (0,52–3,60)	12,871 (5,81–18,63)^a^	2,850 (1,49–5,78)^b^	1,477 (1,14–3,83)^b^	0,06
HIF-1 α (pg/ml)	0,947 (0,6–4,76)	0,53 (0,4–0,50)	0,631 (0,5–1,24)	0,8 (0,3–0,9)	0,164
IL-1ẞ (pg/ml)	168 (83–223)	427 (273–649)^a^	223 (82–273)	238 (148–289)	0,048
IL 6 (pg/ml)	1,607 (0,90–2,78)	4,740 (3,98–6,04)	0,789 (0,53–0,88)^b^	0,719 (0,32–1,14)^b^	0,011
TNF-α (pg/ml)	462 (359–617)	937 (916–1365)	197 (129–389)^b^	336 (304–397)	0,009

*VEGF *vascular endothelial growth factor, *TNF − α *tumor necrosis factor − alpha, *IL − 1β *interleukin − 1 beta, *IL-6 *interleukin − 1, *HIF − 1α *hypoxia-inducible factor − 1 alpha, *G1 *Control group, *G2 *OHSS group, *G3 *OHSS group given cabergoline, *G4 *OHSS group given nifedipine

^*^Kruskal–Wallis

^a^ Compared with G1

^b^ Compared with G2, (*p* < 0.05)

#### Serum HIF-1 α Levels

When comparing G2, G3, and G4 to G1, no statistically significant difference was found (*p* = 0.597), (Table 3).

#### Tissue HIF-1 α Levels

When comparing G2, G3, and G4 to G1, no statistically significant difference was found (*p* = 0.164), (Table 4).

#### Serum IL-1β Levels

Serum IL-1β levels in G2 were found to be statistically significantly higher than those in G1 (*p* < 0.001). When comparing G3 to G2, there was no statistically significant change in IL-1β levels (*p* = 0.307), whereas G4 showed a statistically significant reduction (*p* = 0.021). Nevertheless, there was no discernible statistically significant change between G3 and G4 (*p* = 0.997), (Table 3).

#### Tissue IL-1β Levels

Tissue IL-1β levels in G2 were found to be statistically significantly higher than those in G1 (*p* = 0.043). There was no statistically significant difference in tissue IL-1β levels between G3 (*p* = 0.239) and G4 (*p* = 0.859) compared to G2. Nevertheless, there was no noticeable statistically significant difference between G3 and G4 (*p* = 0.998), (Table 4).

#### Serum IL6 Levels

Serum IL6 levels in G2 were found to be statistically significantly higher than those in G1 (*p* = 0.003). There was no statistically significant change in IL6 levels in G3 (*p* = 0.384) as compared to G2, but there was a statistically significant decrease in G4 (*p* = 0.002). Nevertheless, there was no observable statistically significant difference between G3 and G4 (*p* = 0.547), (Table 3).

#### Tissue IL6 Levels

Tissue IL6 levels in G2 did not differ statistically significantly from those in G1 (*p* = 0.928). In comparison to G2, IL6 levels were statistically significantly lower in G3 (*p* = 0.021) and G4 (*p* = 0.044). G3 and G4 did not, however, differ statistically significantly (*p* = 0.996), (Table 4).

#### Serum TNF-α Levels

Comparing G2 to G1, there was a statistically significant increase in serum TNF-α levels (*p* = 0.002). There was a statistically significant decrease in serum TNF-α levels in G4 (*p* = 0.003), but no statistically significant change in G3 compared to G2 (*p* = 0.093). G3 and G4 did not, however, differ statistically significantly (*p* = 0.994), (Table 3).

#### Tissue TNF-α Levels

Tissue TNF-α levels in G2 did not differ statistically significantly from those in G1 (*p* = 0.928). TNF-α levels in G3 were statistically significantly lower than those in G2 (*p* = 0.007). There was no statistically significant difference between G4 and G3 (*p* = 0.985) or between G4 and G2 (*p* = 0.131), (Table 4).

## Discussion

In this study, we examined how nifedipine affects the suppression of VEGF and proinflammatory cytokines that are crucial to the pathophysiology of OHSS, including HIF-1 α, IL-1β, IL-6, and TNF-α. Our research revealed that nifedipine, like cabergoline, lowers VEGF levels in ovarian tissue and serum. We also demonstrated that nifedipine lowers cytokine levels. We demonstrated that nifedipine can likewise accomplish these effects by blocking the TRPC1 ion channel. The effectiveness of the old medication nifedipine in OHSS is being examined for the first time in our study.

Numerous investigations have demonstrated that VEGF production is decreased when TRPC1 is inhibited [16, 17, 25]. Given that TRPC1 from the TRPC family may be more closely associated with VEGF, which is crucial to the pathophysiology of OHSS, we thought it suitable to investigate its activity in our work. It has been demonstrated that nifedipine inhibits the expression of inflammatory proteins such as IL-1β, IL-6, and TNF-α, as well as TRPM-7, TRPC-1, TRPC-3, and TRPC-6. This lowers VEGF expression and eliminates skin redness and edema [25]. As a result of our investigation, we demonstrated that TRPC1 immunoreactivity rises in OHSS. It was shown that nifedipine dramatically decreased TRPC1 immunoreactivity, whereas cabergoline was unable to suppress it. However, a significant rise in TRPC1 gene expression was observed in comparison to the cabergoline group, whereas a significant decrease in gene expression was observed in the nifedipine group when compared to the control group. This rise can be explained by the fact that gene expression has increased to make up for the tissue's decreased TRPC1 immunoreactivity. By binding to VEGF2 receptors on endothelial cells, increased VEGF brought on by hCG injection promotes angiogenesis and contributes to the pathogenesis of OHSS by raising cytosolic Ca^2+^ concentration and vascular permeability [9, 26]. In our study, the conditions of TRPC1 inhibition on OHSS were examined macroscopically. However, the direct effect of nifedipine on TRPC1 could not be clearly explained by our present findings. A decrease in signal transducer and activator of transcription 3 (STAT3) and NF-κB p65 phosphorylation was reported in TRPC1 siRNA transfected lung epithelial cell line (MLE-12) cells. In contrast, STAT3 and NF-κB phosphorylation were shown to be increased in TRPC1 overexpressing MLE-12 cells compared with the control group. Additionally, loss of TRPC1 has been shown to cause decreased secretion of IL-6, TNF-α, IL-4, IL-10 and IL-1β. These findings support that TRPC1 deficiency may exhibit impaired proinflammatory responses via NF-κB and STAT3. It has been suggested that when TRPC1 is inhibited, the cell cycle is arrested in the S phase, and when TRPC1 is overexpressed, the G2 and M phases are acceleratedThese results suggest that TRPC1 may affect the cell cycle through activation of the STAT3/NF-κB pathway [27]. In our study, nifedipine may have also shown its TRPC1 inhibition effect on the cell cycle. Further studies should be designed. Rats in our study were given hCG to induce ovulation [26]. It has been demonstrated that endothelial cells expressing TRPC1 enhance VEGF-induced Ca^2+^ influx [28]. High calcium permeability, nonselective cationic channels are present in the TRPC1 protein. Many human tissues and cell types express these channels in large quantities [29]. Drugs to treat cancer, epilepsy, pain, arthritis, and heart conditions may therefore be developed with these ion channels as prospective targets [30]. Şanlı et al. [31] found that elevated TRPM2 and CHRM1 [6] immunoreactivity in hyperstimulated rat ovaries may contribute to the pathophysiology of OHSS as a cause or effect of congestion and edema in an experimental investigation examining ion channel activities in OHSS. Following a single dosage or prolonged cabergoline administration, Atilgan et al. [21] demonstrated a substantial decrease in VEGF-2 levels in ovarian tissues. In moderate or severe-risk women, cabergoline has been shown to lower the risk of OHSS [32]. Our research showed that the OHSS group had much higher blood and tissue VEGF levels than the control group, and that cabergoline and nifedipine effectively reduced serum and tissue VEGF levels to a comparable degree to the OHSS group. In one study, nifedipine decreased the mRNA levels of VEGF and TRPC-6 in skin tissue, according to RT-PCR analysis [25]. Another study found that VEGF secretion was decreased when Ca^2+^ channels were inhibited with nifedipine (10–5 M), whereas VEGF secretion was increased when L-type channels were stimulated [33]. The recent studies'findings might also help to explain why our study's nifedipine treatment reduced the VEGF levels in rats with OHSS. However, research on nifedipine has revealed that, in addition to its traditional mode of action, the drug's pleiotropic effects cause the synthesis of VEGF, the most powerful angiogenic factor. By increasing the distance between cells by its vasodilation action and promoting endothelial cell migration, nifedipine may also promote microvascular angiogenesis [34, 35]. Nevertheless, nifedipine's molecular effects have also been demonstrated to decrease cell death in hypoxic cells [18]. By controlling microcirculation, boosting regional blood flow, and enhancing tissue tolerance to ischemia, nifedipine applied topically and systemically to ischemic skin flaps in rats has been demonstrated to decrease necrotic areas [36, 37]. Given that nifedipine may reorganize microcirculation and tissue homeostasis differently, and because the increase in VEGF in OHSS may be mediated by a variety of different mediators, the nifedipine group in our study had lower VEGF levels. However, it has been demonstrated that TRPC1 alleviates edema and redness in the foot skin and lowers VEGF expression in the skin of chilblain rats [25]. In our study, we showed that nifedipine inhibits the TRPC1 channel, which reduces inflammation and VEGF production in OHSS. However, further molecular studies are needed to fully elucidate how this inhibition occurs at the molecular level. Nifedipine's suppression of inflammation and VEGF production via TRPC1 inhibition can be explained by the findings in similar studies. It has been shown in vitro that nifedipine can activate TRPC channels, especially TRPC1, leading to Ca2 + influx into myometrial cells, but this effect can only occur at high drug concentrations (> 10 − 6 M). Despite effective downregulation of genes encoding all channels tested, nifedipine-induced Ca2 + influx was shown to be significantly reduced only in cells where TRPC1 expression was downregulated. Therefore, it was suggested that the rapid increase in intracellular Ca2 + observed in the pregnant human myometrial 1–41 (PHM1-41) cells in response to nifedipine may be due to a direct blocking effect of nifedipine on TRPC1 [36]. Our findings are in line with those of this study. The most significant stimulant for VEGF synthesis is hypoxia [38]. HIF-1, which attaches to the VEGF gene promoter, is activated to deliver this stimulation [39]. In their experimental investigations, Pala et al. [40] showed how VEGF contributes to the development of hypoxia in OHSS. Through the transcription of several hypoxia response genes, HIF-1α controls angiogenesis [41]. In mice suffering from myocardial infarction (MI), it has been demonstrated that HIF-1α directly increases the expression of TRPC1. This has been shown to improve heart function following MI by promoting angiogenesis [17]. There was no discernible difference between the groups in our study when serum and tissue HIF-1 α levels were evaluated across all groups. The findings of the aforementioned research are contrary to these findings. There may not have been enough hypoxic conditions in the rat ovaries where we produced OHSS, and additional compensatory mechanisms might have been at work.

A significant rise in systemic inflammatory cytokines and vasoactive substances, including VEGF, interleukin-1β, IL-6, and TNF-α, in serum, follicular, and ascitic fluid may be the etiology of OHSS [6, 42]. Moreover, hCG causes ovulation, permits a rise in the number of oocytes, and starts inflammatory secretion in human granulosa cells, all of which contribute to the systemic inflammatory response during OHSS [42, 43]. Serum levels of IL-1β, IL-6, and TNF-α were higher in our OHSS group than in the control group, according to our study. We demonstrated that nifedipine markedly decreased TNF-α, IL-1β, and IL-6 levels. There was no difference between the OHSS, cabergoline, and nifedipine groups, despite the fact that tissue IL-1β levels were higher in the OHSS group than in the control group. In contrast to the OHSS group, the nifedipine and cabergoline groups had lower levels of IL-6. Comparing the cabergoline and OHSS groups, the cabergoline group's tissue TNF-α levels were noticeably lower. There was no significant difference between the cabergoline and nifedipine groups when compared. Numerous inflammatory responses of vascular endothelial cells are regulated by the classical immune inflammatory cytokines TNF-α, IL-1β, and IL-6 [44]. By lowering the expression of TRP family proteins, nifedipine has been demonstrated to decrease the cytokine production of IL-1β, IL-6, TNF-α, and VEGF [25]. In our investigation, we demonstrated that nifedipine had an anti-inflammatory impact by significantly lowering the serum levels of inflammatory cytokines, including TNF-α, IL-1β, and IL-6, in rats with OHSS. We believe that nifedipine's anti-inflammatory action stems from a decrease in TRPC1 channel activation. One impact of nifedipine that may help prevent the development of OHSS is its anti-inflammatory properties. In many cell types, TNF-α-induced secretory activities rely on Ca^2+^. For instance, it has been demonstrated that nifedipine inhibits the release of IL-6 when it blocks the lung's endothelial L-type CaV channels. Nifedipine has been shown to have comparable anti-inflammatory effects in organs other than the lung [45]. In human chondrocytes, nifedipine has been demonstrated to suppress oxidative stress and the release of inflammatory mediators [46]. By showing for the first time the anti-inflammatory benefits of nifedipine in a rat OHSS model caused by hCG, our findings advance our understanding in this field. Matrix metalloprotein (MMP)−13, interleukin (IL)−1β, IL-6, tumor necrosis factor (TNF)-α, cyclooxygenase (COX)−2, and inducible nitric oxide (NO) have all been demonstrated to be inhibited by nifedipine [46]. Another study found that mucus volume decreased when TRPC1 expression was blocked. This has been linked to lower proinflammatory cytokines such IL-4, IL-1β, and TNF-α as well as down-regulated inflammatory pathways, especially the STAT3/NF-κB signaling pathway [27]. Through the TLR4/TRPC1/NF-κB signaling pathway, TRPC1 has also been shown to contribute to the inflammatory response to bacterial infection [47].

In guinea pigs with asthma, expression of the TRPC1 channel has also been linked to the development of chronic airway inflammation [48]. The reduction in inflammatory cytokines in the nifedipine group is probably due to the drug's superior anti-inflammatory qualities [49]. According to recent research, nifedipine's main function in the uterus is thought to be to stop calcium-dependent muscle contractions, but there is also evidence that it may have cytoprotective and anti-inflammatory effects outside of pregnancy by blocking calcium channels in non-pregnant tissues [48, 50]. Nevertheless, it has been demonstrated that nifedipine has no effect on the release of inflammatory cytokines or the expression of inflammatory markers from human myometrial cells and tissues. According to this study, the inhibition of smooth muscle contractions by nifedipine was not mediated by the control of proinflammatory cytokines [51]. Some of the variations in serum and tissue inflammatory cytokine levels observed in our investigation may be explained by the findings of this study. However, our study used serum and ovarian tissue, whereas their study used uterine tissue. The differences between species should also be considered.

The limitation of our study include the fact that it was an experimental investigation and that the outcomes of experiments cannot be used to replicate human results, as well as the fact that it was carried out in a small population because of the limited number of rats. In our study, the short-term effects of nifedipine and cabergoline were investigated, and the long-term effects of both drugs on OHSS could not be evaluated. In particular, the possible side effects of long-term inhibition of TRPC1 and the effect on other visible effects were not investigated, which is one of the limitations of our study. In our study, only the severe OHSS model was used. Examining the mild and moderate OHSS models could reveal how the effects of drugs change in these cases and could be more meaningful for clinical practice. This constitutes another limitation of our study. Our study's strengths are that it is the first to examine the activities of TRPC1 and cytokines in OHSS, the first to examine the effects of cabergoline and nifedipine on these parameters, and the first to examine the pathophysiology of OHSS from a different angle. These findings may serve as models for future research that can close this gap in the literature. In conclusion, nifedipine inhibits the production of proinflammatory cytokines such IL-1β, IL-6, and TNF-α, as well as VEGF, most likely via blocking TRPC1 channel activation. This lowers the development of OHSS.
